# Maternal and Neonatal Outcomes of Cervical Colonization in Adolescent Pregnancies: A Brief Report

**DOI:** 10.3390/pediatric17020036

**Published:** 2025-03-12

**Authors:** Maisa Manasar-Dyrbuś, Jakub Staniczek, Rafał Stojko, Piotr Gibała, Cecylia Jendyk, Ewa Winkowska, Kacper Niziński, Diana Sieroszewska, Aleksander Sieroszewski, Agnieszka Drosdzol-Cop

**Affiliations:** 1 Clinical Department of Gynecology, Obstetrics and Gynecological Oncology, The Medical University of Silesia in Katowice, Markiefki 87, 40-211 Katowice, Poland; maisamanasar@gmail.com (M.M.-D.);; 2 Department of Gynecology, Obstetrics, Gynecological Oncology, Pediatric and Adolescent Gynecology, Bonifraters’ Medical Center, Markiefki 87, 40-211 Katowice, Poland

**Keywords:** adolescent pregnancy, cervical colonization, adolescent, high risk pregnancy

## Abstract

Objective: This study examines early neonatal adaptation and birth complications in adolescents with term pregnancies who had positive cervical canal cultures upon hospital admission. Methods: This retrospective study analyzed data from 1 January 2015 to 15 November 2024. Conducted at Bonifraters Medical Center in Katowice, Poland, the study included 267 individuals, with 178 over the age of 19 and 89 under the age of 19. Results: Adolescents exhibited significantly higher rates of positive GBS cultures in the third trimester (62.92% vs. 38.20%; *p* < 0.001) than older individuals. Neonates of adolescent mothers experienced increased congenital pneumonia (7.87% vs. 1.12%; *p* = 0.012) and antibiotic administration (10.11% vs. 2.81%; *p* = 0.026). Lactation failure was markedly higher in adolescent mothers (10.11% vs. 1.12%; *p* = 0.002). Adolescents also showed more postpartum blood loss (median: 250 mL vs. 200 mL; *p* < 0.001) and more extended hospital stays (median: 3 vs. 2 days; *p* = 0.002). Neonatal anthropometric measures revealed shorter body lengths in the adolescent group (median: 53 cm vs. 54 cm; *p* = 0.003). Conclusions: Adolescent pregnancies are associated with significantly higher rates of complications and adverse neonatal outcomes, especially infectious causes. Although our study results are preliminary, it appears that chronic GBS colonization in pregnant adolescents may impact pregnancy and newborn outcomes.

## 1. Introduction

Adolescent pregnancies present a significant public health challenge due to their association with increased maternal and neonatal risks. Numerous studies have demonstrated that younger maternal age is linked to higher rates of obstetric complications, including uterine atony, postpartum hemorrhage, and perinatal infections, as well as adverse neonatal outcomes [1,2,3].

Adolescent pregnancies are notably more prone to infections, particularly Group B *Streptococcus agalactiae* (GBS) colonization, which often requires early antibiotic intervention to mitigate these risks [1,2]. Furthermore, systematic reviews and meta-analyses have identified an elevated risk of fetal abnormalities associated with infections in neonates born to adolescent mothers, highlighting the importance of enhanced prenatal diagnostics [4,5].

These factors contribute to disparities in care and underscore the urgent need for multidisciplinary approaches to improve maternal and neonatal health outcomes [4,6,7]. National guidelines, such as those issued by the Polish Society of Gynecologists and Obstetricians, emphasize the importance of early diagnostic strategies, psychological support, and comprehensive antenatal care to address these disparities [6].

This study builds upon existing evidence by retrospectively analyzing early neonatal adaptation and birth complications in adolescents with term pregnancies and positive cervical canal cultures upon hospital admission.

## 2. Methodology

This retrospective study aims to investigate early neonatal adaptation and birth complications in adolescents with term pregnancies and positive cervical canal cultures, comparing these outcomes with those observed in pregnancies among older women. According to the hospital’s standard protocol, every pregnant admitted undergoes a bacterial swab of the cervical canal. From 1 January 2015, to 15 November 2024, all pregnant individuals beyond 37 weeks of gestation with confirmed cervical colonization at the time of hospital admission who delivered at the Department of Gynecology, Obstetrics, Oncological Gynecology, Pediatric, and Adolescent Gynecology at the Bonifraters Medical Center in Katowice, Poland, were included in this study, were during the study period, a total of 17,380 individuals gave birth. To minimize bias, individuals with incomplete medical records were excluded. The study cohort was divided into two primary groups: adolescents (≤19 years old) and a matched control group consisting of individuals > 19 years old. In total, 267 individuals met the inclusion criteria, including 89 adolescents (≤19 years old) and 178 individuals in the control group (>19 years old). The dataset comprises hospital medical records. The data were stored using Mediqus software (version 4.052.J.3; license WMS Mediqus Szpital; Gabos, Poland) and KS-Medis software (version 2024.02.3.0; license 2142PI01.00; Kamsoft, Poland). All data were anonymized and processed by relevant regulations. Independent researchers extracted the data from the system. Additionally, two researchers (M.M-D. and J.S.) independently analyzed and extracted the anonymized data.

## 3. Statistical Analyses

Statistical analysis was performed using R Studio (version 4.4). The control group was extracted from the database at a 2:1 ratio to the study group using propensity score matching. Participants in the control group were matched based on similarities in gestational age, premature rupture of membranes (PROM), and parity. A descriptive analysis of variables was presented in tables created using Microsoft Excel (Microsoft 365). The normality of variable distributions was assessed using Q-Q plots generated in R Studio.

Relationships between variables were evaluated using the chi-square test for categorical variables, the Student’s *t*-test for continuous variables with distributions approximating normality, and the Wilcoxon test for continuous variables with non-normal distributions. A significance level of *p* < 0.05 was used as the threshold for statistical significance.

For categorical variables, odds ratios (OR) and their 95% confidence intervals (CI) were calculated to assess the strength of associations. For sparse data, a correction was applied to avoid division by zero. Key results were visualized using forest plots to interpret the findings.

## 4. Results

### 4.1. General Characteristics

The study group included 267 participants aged 15–46 years, with a median age of 24 (IQR: 19–27) and a mean age of 24.39 ± 6.63 years. Most (84.27%) were in their first pregnancy. The mean gestational age at delivery was 38.88 weeks (median: 39 weeks, IQR: 38–40), and the mean hospital stay was 2.85 days (median: 2 days, IQR: 2–3). Approximately 46.44% tested positive for GBS colonization in the third trimester. Per Polish guidelines, screening occurs at 35–37 weeks via vaginal and rectal swabs.

Microbiological findings revealed that *Candida albicans* was the most frequently isolated microorganism, identified in 66.29% of cases. Antibiotic therapy was widely utilized; 48.31% received antibiotics upon admission, primarily ampicillin (69.7%), while 29.96% continued antibiotic treatment after discharge, predominantly with amoxicillin (88.8%).

Peripartum complications were noted in a substantial proportion. Episiotomy was performed in 21.35% of cases, and postpartum hemorrhage occurred in 5.24%, with an average blood loss of 249.4 mL. Neonatal complications included congenital pneumonia (3.37%) and respiratory distress requiring nasal continuous positive airway pressure (nCPAP) in 53.85% of cases. The mean neonatal birth weight was 3304.7 g (median: 3200 g, IQR: 3040–3595), and the mean length was 53.8 cm (median: 54 cm, IQR: 52–56). The mean Apgar score at 1 min post-delivery was 9.82 (median: 10, IQR: 10–10).

Laboratory findings revealed white blood cell (WBC) counts at admission ranging from 1.63 to 25.2 × 10^3^/μL (median: 12 × 10^3^/μL, IQR: 10.48–14 × 10^3^/μL, mean: 12.44 ± 3.29 × 10^3^/μL) and C-reactive protein (CRP) levels ranging from 0.1 to 74.32 mg/L (median: 5 mg/L, IQR: 2.27–11.49, mean: 9.48 ± 12.73 mg/L). Neonatal blood gas analyses, available for a subset of 18 neonates, revealed a median pH of 7.32 (IQR: 7.28–7.38). Procalcitonin levels, measured in a smaller subgroup (n = 8), ranged from 0.3 to 10.22 ng/mL, with a median of 1.25 ng/mL (IQR: 0.5–2, mean: 2.25 ± 3.31 ng/mL).

### 4.2. Comparison Between Groups

GBS colonization in the third trimester was significantly more frequent in women aged ≤19 years compared to those aged >19 years (62.92% vs. 38.20%, *p* < 0.001). Participants aged ≤19 years had higher, though statistically nonsignificant, rates of *Candida albicans* (*p* = 0.131), *Enterococcus faecalis* (*p* = 0.065), and *Escherichia coli* (*p* = 0.065).

Antibiotic therapy was used significantly more often in the ≤19 groups (67.42% vs. 38.76%, *p* < 0.001). Neonates born to mothers aged ≤19 years had significantly higher rates of antibiotic administration (10.11% vs. 2.81%, *p* = 0.026) and congenital pneumonia (7.87% vs. 1.12%, *p* = 0.012). Lactation failure was also significantly more frequent in the ≤19 group (10.11% vs. 1.12%, *p* = 0.002).

Regarding delivery complications, uterine atony (*p* = 0.031) and postpartum hemorrhage (*p* = 0.099) were more common in the ≤19 group. Conversely, episiotomy (*p* < 0.001) and postpartum uterine curettage (*p* = 0.012) were significantly less frequent in this group. Postpartum blood loss was also higher in the ≤19 group (*p* < 0.001). Additionally, hospital stays were longer in the ≤19 group, with a median of 3 days (IQR: 2–3) compared to 2 days (IQR: 2–3) in the >19 group (*p* = 0.002).

Anthropometric differences in neonates included shorter median lengths in the ≤19 groups (53 cm, IQR: 51–55) compared to the >19 groups (54 cm, IQR: 52–56; *p* = 0.003). Hematological differences were also observed, with significantly lower lymphocyte counts in the ≤19 groups (*p* = 0.001).

**Figure 1:**
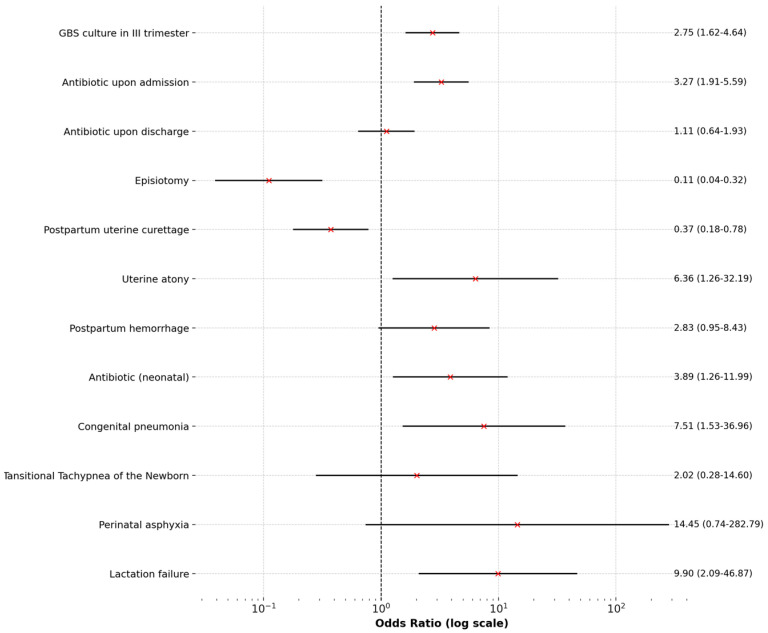
Forest Plots of the Odds Ratios (OR) with 95% confidence intervals for qualitative variables in adolescents aged ≤19.

**Figure 2:**
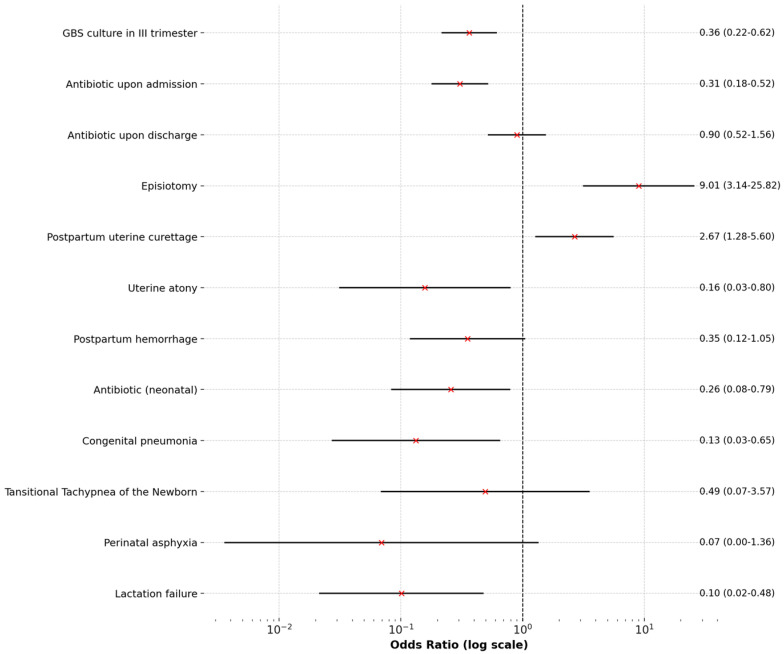
Forest plots of odds ratios (OR) with 95% confidence intervals for qualitative variables in women aged >19 years.

**Figure 3:**
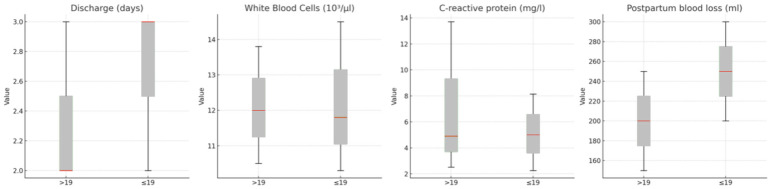
Comparison of quantitative variables between groups in terms of pregnancy characteristics between groups.

**Figure 4:**
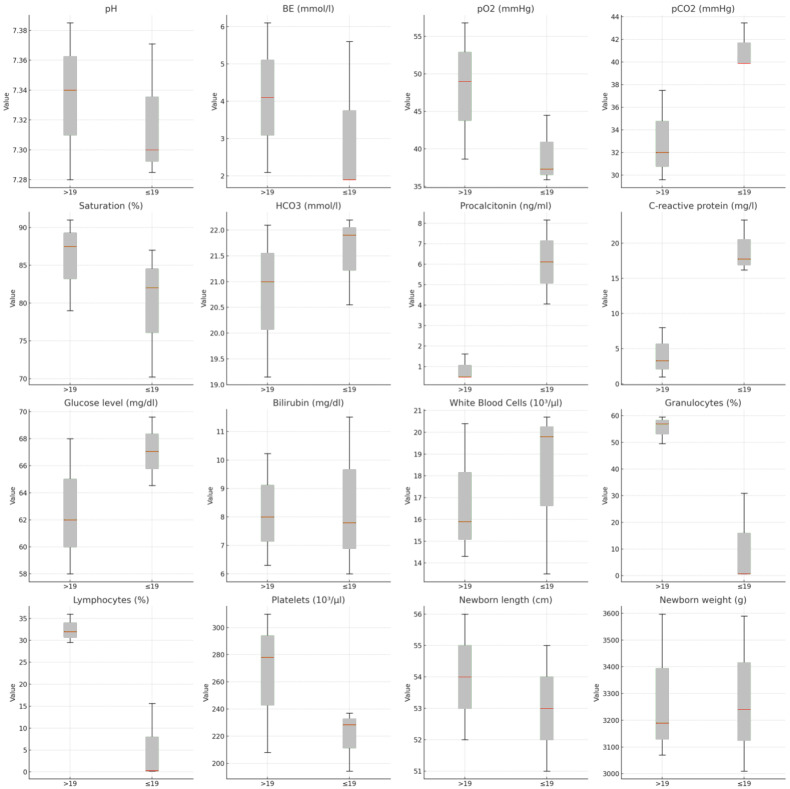
Comparison of quantitative variables between groups in terms of newborn outcomes between groups.

Table 1 presents a comparison of qualitative variables, while Figure 1 and Figure 2 display Forest Plots of Odds Ratios (OR) with 95% confidence intervals, illustrating differences in qualitative variables between adolescents aged ≤19 and women aged >19. Figure 3 and Figure 4 compare quantitative variables between the two groups.

## 5. Discussion

Adolescent pregnancy continues to pose a significant public health challenge, with numerous adverse consequences for both the mother and child [8]. The group aged ≤19 demonstrated a distinct risk profile compared to older groups. This finding aligns with existing evidence highlighting the vulnerabilities associated with adolescent pregnancies. In our study, younger individuals were significantly more frequently colonized by Group B *Streptococcus agalactiae* in the third trimester of pregnancy (62.92% vs. 38.20%, *p* < 0.001). This result is consistent with the observations of Akoh et al. [2], who reported a higher prevalence of bacterial colonization and infections in adolescent pregnancies, particularly those involving GBS. Young women exposed to maternal infections are at a heightened risk for congenital fetal anomalies [6]. In our study, cervical colonization necessitated more frequent antibiotic therapy during hospitalization (67.42% vs. 38.76%, *p* < 0.001), with clindamycin being a notable post-discharge choice among younger (*p* = 0.023). These therapeutic interventions, while essential, underline the increased infectious risks in this demographic, as also emphasized by Çift et al. [5], who advocated for regular microbial screening and prompt management.

In the context of this issue, socioeconomic factors emerge as a critical determinant, as lower socioeconomic status is strongly associated with higher risks of complications, including postpartum hemorrhage, operative deliveries, and preterm births [9]. Additionally, the late initiation and inadequate utilization of antenatal care, which are often observed among adolescent mothers, further exacerbate the likelihood of poor obstetric outcomes [10].

Our study’s ≤19 age group also exhibited a significantly higher risk of peripartum complications. Uterine atony occurred more frequently (6.74% vs. 1.12%, *p* = 0.031), along with increased postpartum blood loss (median 250 mL vs. 200 mL, *p* < 0.001). These findings align with those of Staniczek et al. and Diabelková et al., who identified common complications in adolescent pregnancies, such as uterine atony and excessive blood loss, which often contribute to prolonged hospital stays [1,7]. Our cohort’s median hospitalization duration was significantly longer for younger individuals (3 days vs. 2 days, *p* = 0.002), reflecting the more significant clinical burden. Similarly, Staniczek et al. [1] underscored prolonged hospitalizations as an indicator of heightened peripartum risk among adolescents.

Neonatal outcomes in the ≤19 age group further reinforced these concerns. Neonates born to younger mothers faced significantly higher risks of congenital pneumonia (7.87% vs. 1.12%, *p* = 0.012) and were more likely to receive antibiotics postpartum (10.11% vs. 2.81%, *p* = 0.026). These findings echo those of Çift et al. [5] and Azevedo et al. [3], both of whom reported increased neonatal morbidity, including respiratory complications, in infants of adolescent mothers. Additionally, our study identified a significant difference in neonatal length (*p* = 0.003). However, birth weight did not differ significantly. We observed a significantly higher prevalence of lactation difficulties among adolescents than adults (10.11% vs. 1.12%, *p* = 0.002). These challenges can have negative consequences for both the mother and the child. For the infant, inadequate breastfeeding may lead to suboptimal nutritional intake, impaired immune protection, and hindered growth and development. For the mother, lactation difficulties can contribute to increased stress, feelings of inadequacy, and a higher risk of postpartum depression [6]. Moreover, these issues may disrupt early mother-infant bonding and interfere with the infant’s physiological regulation, particularly in preterm infants, where early maternal contact and nutrition are critical [11].

Laboratory findings revealed significantly lower lymphocyte counts in the ≤19 age group (*p* = 0.001). Although elevated procalcitonin levels in neonates did not reach statistical significance in our study, they suggest potential differences in immune response and inflammatory predisposition.

Adolescent pregnancies are associated with significantly higher complications and adverse neonatal outcomes. These findings, supported by extensive literature, including systematic reviews and national recommendations [1,3,4], underscore the need for individualized perinatal care for this demographic. However, it is essential to emphasize that our study’s results are preliminary. Further research is necessary to validate these observations, explore underlying mechanisms, and guide the development of targeted interventions for improving maternal and neonatal outcomes in this population, particularly concerning the potential reduction of GBS colonization in adolescents.

## 6. Clinical Implications

This study highlights the need for tailored management strategies in adolescent pregnancies, given the heightened maternal and neonatal risks observed in this population. The increased incidence of congenital pneumonia and postpartum antibiotic administration, along with significantly higher rates of postpartum hemorrhage and prolonged hospital stays among adolescent mothers, necessitate enhanced obstetric surveillance and individualized postpartum care plans to manage these complications effectively. Furthermore, the higher prevalence of lactation difficulties in this group highlights the need for structured breastfeeding support programs to optimize neonatal nutrition and maternal well-being.

## Figures and Tables

**Table 1:** Comparison of qualitative variables between groups.

Variable	>19		≤19	*p*-Value
n	178		89	
GBS positive culture in III trimester	68 (38.20%)		56 (62.92%)	<0.001
Cultured pathogens				
*Candida albicans*	112 (62.92%)		65 (73.03%)	0.131
*Candida glabrata*	14 (7.87%)		4 (4.49%)	0.437
*Candida tropicalis*	1 (0.56%)		0 (0.00%)	1
*Candida crusei*	0 (0.00%)		1 (1.12%)	0.723
*Streptococcus agalactiae*	62 (34.83%)		32 (35.96%)	0.964
*Staphylococcus aureus*	20 (11.24%)		8 (8.99%)	0.724
*Enterococcus faecalis*	0 (0.00%)		3 (3.37%)	0.065
*Escherichia coli*	0 (0.00%)		3 (3.37%)	0.065
Antibiotic therapy				
Antibiotic	69 (38.76%)		60 (67.42%)	<0.001
Type of antibiotic	Amoxicillin	3 (4.29%)	7 (11.11%)	0.003
	Ampicillin	57 (81.43%)	33 (52.38%)	
	Clindamycin	10 (14.29%)	20 (31.75%)	
	None	0 (0.00%)	3 (4.76%)	
Antibiotic upon discharge	52 (29.21%)		28 (31.46%)	0.813
Antibiotic upon discharge	Amoxicillin	51 (92.73%)	24 (85.00%)	0.023
	Clindamycin	4 (7.27%)	4 (25.00%)	
Labor complications				
Episiotomy	53 (29.78%)		4 (4.49%)	<0.001
Postpartum uterine curettage	45 (25.28%)		10 (11.24%)	0.012
Uterine atony	2 (1.12%)		6 (6.74%)	0.031
Postpartum hemorrhage	6 (3.37%)		8 (8.99%)	0.099
Neonatal outcomes				
nCPAP	4 (100.00%)		3 (33.33%)	0.105
Antibiotic	5 (2.81%)		9 (10.11%)	0.026
Congenital pneumonia	2 (1.12%)		7 (7.87%)	0.012
Tansitional Tachypnea of the Newborn	2 (1.12%)		2 (2.25%)	0.859
Perinatal asphyxia	0 (0.00%)		3 (3.37%)	0.065
Lactation failure	2 (1.12%)		9 (10.11%)	0.002

## Data Availability

The original contributions presented in this study are included in the article. Further inquiries can be directed to the corresponding author.
